# Attachment style, thought suppression, self-compassion and depression: Testing a serial mediation model

**DOI:** 10.1371/journal.pone.0245056

**Published:** 2021-01-14

**Authors:** Clara V. Murray, Juno Irma-Louise Jacobs, Adam J. Rock, Gavin I. Clark

**Affiliations:** 1 School of Psychology, University of New England, Armidale, Australia; 2 School of Psychology, Newcastle University, Newcastle upon Tyne, United Kingdom; Unviersity of Sheffield, UNITED KINGDOM

## Abstract

Attachment anxiety has been consistently linked with increased vulnerability to depression, and hyperactivating emotion regulation strategies (e.g., rumination) have been shown to mediate this relationship. Investigations of mediators of the attachment avoidance to depression relationship have yielded inconsistent findings, and the nature of this relationship remains to be clarified. There is evidence to suggest that the constructs of thought suppression and self-compassion are associated with attachment avoidance and also with depressive symptomology. In order to further clarify the nature of this relationship, the present study tested a serial mediation model, whereby it was hypothesised that thought suppression and self-compassion were serial mediators of the relationship between attachment avoidance and depression. One hundred and forty-eight participants completed an online composite questionnaire consisting of the Experiences in Close Relationships-Revised Questionnaire, the White Bear Suppression Inventory, the Self-Compassion Scale, and the Depression Anxiety and Stress Scale-21. Initial results supported the hypothesised serial mediation model (Model A); that is, higher attachment avoidance predicted higher thought suppression, higher thought suppression predicted lower levels of self-compassion and lower levels of self-compassion predicted higher depression. However, this model was no longer significant following the inclusion of attachment anxiety as a covariate within the post-hoc analysis. A second, post-hoc serial mediation model was tested (Model B), with the only difference being that attachment anxiety replaced attachment avoidance as the independent variable. This model was significant, with and without the inclusion of attachment avoidance as a covariate. The study provides evidence for the central role of thought suppression and self-compassion as mechanisms underlying the relationship between insecure attachment and depression, and indicates that these factors operate in opposing directions. The findings are discussed in terms of explicating some of the processes through which insecure attachment confers vulnerability to depression. The implications of the observed degree of shared variance between the two attachment dimensions suggests these constructs may be more appropriately considered overlapping, rather than orthogonal.

## Introduction

### Adult attachment

Attachment theory has provided a useful framework for understanding individual vulnerability to psychological distress and, in particular, to depression [1, 2]. Attachment theory posits that individuals develop cognitive-emotional models (internal working models) of self and others based on early caregiver availability and responsiveness [3]. These models provide the foundation for adult attachment patterns, and have been proposed to influence the manner in which individuals regulate emotion throughout the lifespan [2].

Adult attachment style has been defined and measured in relation to two orthogonal dimensions: attachment avoidance and attachment anxiety [2]. Individuals whose attachment style reflects heightened levels of attachment anxiety and/or attachment avoidance would be considered to have an insecure attachment; whilst lower levels of attachment anxiety and attachment avoidance are reflective of secure attachment [3].

Attachment avoidance has been linked with early interactions with caregivers who were unresponsive, unavailable and/or abusive [4]. Attachment theory proposes that through these early interactions, individuals learn to distrust the ability of other people to provide support, and, in turn, develop strategies to down-regulate the felt sense of need for care/support [5, 6]. These so-called deactivating strategies include compulsive self-reliance, avoidance of intimacy and close relationships and the suppression of attachment-related thoughts and emotions [7].

Experiences with inconsistently available caregivers contribute to attachment anxiety, and the development of hyperactivating attachment strategies. These typically involve increasing the salience of distress to gain the support of significant others [3, 4].

### Adult attachment and depression

Major Depressive Disorder (MDD) is a common mood disorder characterised by low mood and/or anhedonia, and a symptom profile which can include disrupted sleep and appetite, reduced concentration and feelings of worthlessness and guilt [8]. Depression is the leading cause of disability worldwide, with prevalence rates for MDD estimated at 10.4%, or over one million Australians meeting criteria for MDD, during any given year [9]. For individuals with a diagnosis of MDD, there is often an episodic or relapsing/remitting course. Within the general population, depressive symptomatology is experienced on a continuum of severity and intra-individual fluctuations in symptoms are common. As a result, there has been interest in explicating factors which influence the development and exacerbation of depressive symptoms, which is also the focus of the present study. The term ‘depression’ will be used here to refer to the broad continuum of depressive symptomatology, as opposed to MDD [10].

A wide body of research supports a direct positive relationship between insecure adult attachment styles and depression [11–15]. Attachment anxiety has been consistently linked with depression in a variety of studies, including correlational, longitudinal and prospective studies [2, 16, 17]. In contrast, the findings regarding the relationship between attachment avoidance and depression have been mixed. A recent meta-analysis concluded that there is evidence of a significant link between insecure-preoccupied (or anxious) attachment (as measured by the Adult Attachment Interview) and depressive symptoms, but that findings regarding insecure-dismissing attachment (or attachment avoidance) and depressive symptoms was mixed [11]. However, the reported results of the meta-analysis indicate that when insecure-dismissing (or avoidant) attachment was coded as a dimensional rather than categorical variable, individuals with insecure-dismissing attachment had elevated levels of depressive symptoms relative to individuals with a secure attachment style. Taking a dimensional approach, Zheng and colleagues [15]’ meta-analytic study yielded evidence of a strong association between attachment anxiety and depression and a weak association between attachment avoidance and depression.

In summary, research suggests that attachment avoidance impacts upon depressive symptoms [11, 15]. From a theoretical perspective, this association would be expected, given that attachment avoidance reflects a set of negative beliefs and expectations about the availability and willingness of others to provide help or care when this is needed [18]. These negative expectations lead individuals to employ counter-productive intra-psychic and interpersonal strategies in times of distress or need (e.g., deactivating strategies, distancing and withdrawal). Such strategies are typically less effective in down-regulating negative emotions and eliciting support, leading to the confirmation of depressogenic core beliefs about the self, others and the future (e.g., “I’m unworthy”, “no one will ever be there for me”, “in the end, I’m alone”). A wide body of research has demonstrated that the relationship between attachment anxiety and depression is mediated by emotion regulation strategies which would be conceptualised as hyperactivating strategies (see [17] for a review). Conversely, the nature of the relationship between attachment avoidance and depressive symptoms, and what variables may contribute to this relationship, remains to be understood. Furthermore, given that elevated attachment avoidance has been associated with poorer therapeutic outcomes [19], identifying the mechanisms linking attachment avoidance to depression is an important step in targeting interventions and improving therapeutic outcomes. The present study, therefore, examined the relationship between attachment avoidance and depression.

### Emotion regulation, thought suppression and attachment avoidance

Emotion regulation is a multidimensional construct that is operationalised and measured in a number of ways [20]. Emotion regulation has been defined as the collective ability to understand and accept emotional experiences, to employ adaptive strategies to manage emotions, to act in accordance with goals when experiencing negative emotions, and to control impulses [21]. Attachment style is understood to influence the selection of emotion regulation strategies [4]. For example, individuals with an avoidant attachment style may develop deactivating emotion regulation strategies such as minimising the experience and expression of emotions [4, 22]. These strategies are initially functional, helping to maintain proximity to an attachment figure while minimising attachment-related stress [22]. However, chronic over-use of these strategies, particularly in reciprocal adult relationships, is considered maladaptive due to the potential for negative long-term consequences. Examples of maladaptive emotion regulation strategies include suppression [23], experiential avoidance [24] and rumination [25]. Maladaptive emotion regulation has been identified as among the most important mediators linking attachment avoidance to depression [12, 17].

Thought suppression involves active attempts to inhibit unwanted thoughts which may provoke negative emotions [26, 27]. Thought suppression has been linked to increased rates of depression [28]. However, longitudinal studies suggest that thought suppression may interact with life stress, predicting higher rates of depression under conditions of high stress only [29]. Studies of the general population indicate that attempts to suppress thoughts typically result in a heightened accessibility of suppressed thoughts (e.g., a rebound effect) together with increases in emotional and physiological arousal [27].

Thought suppression has been linked with attachment avoidance as a strategy for de-activating the attachment system [30]. Fraley and Shaver [31] demonstrated that, in contrast to the general population, individuals with elevated attachment avoidance do not experience rebound effects when engaging in thought suppression. Specifically, during the suppression condition, these participants did not exhibit elevated galvanic skin responses. The authors interpreted this finding as reflecting successful and complete deactivation of the attachment system and disengagement from attachment-related distress, rather than simply masking or under-reporting underlying distress. In Mikulincer, Dolev, and Shaver’s [32] subsequent experimental study, participants high in attachment avoidance did not experience rebounding of suppressed separation-related thoughts under conditions of low cognitive load. However, for these participants, high cognitive load was associated with reduced ability to suppress separation-related thoughts and increased likelihood of activating negative self-representations. The ability to completely suppress thoughts and re-direct attention elsewhere may be effective in reducing distress under conditions of low stress or load, but less so as demands increase [29, 32]. Additionally, in the context of couple relationships, suppression of attachment-related distress is likely to negatively impact on the development of intimacy and bonding over time [33], and is, therefore, considered a maladaptive emotion regulation strategy.

A systematic review of studies examining attachment orientation, emotion regulation and depression yielded mixed evidence regarding the mediating role of deactivating strategies in the relationship between attachment avoidance and depression [17]. Four of the included studies reported that deactivating strategies significantly mediated the relationship between attachment avoidance and depression in adult samples [34–37]. Brenning and colleagues [38] reported that suppression mediated the relationship between attachment avoidance and depression in adolescents in the second of their two studies. However, three other included studies did not find evidence of significant mediation effects for deactivating strategies [38]. No studies in the review directly explored the relationship between attachment anxiety and suppression. Attachment anxiety is more typically associated with use of hyperactivating strategies [38–40]. However, one experimental study reported that attachment anxiety was associated with difficulties repressing negative cognition and affect [37].

Based on the above, for individuals with elevated attachment avoidance, the use of deactivating strategies has not emerged as a consistent predictor of fluctuations in depressive symptoms. One possible interpretation of the inconsistent findings across studies is that, in the context of attachment avoidance, chronic use of deactivating strategies may potentially contribute to depression in more complex ways, i.e., via more than one intermediary process. The present study examined the possibility that chronic use of thought suppression may interfere with awareness of, and engagement with, emotional distress. As demonstrated by Fraley and Shaver’s [31] experimental study, avoidant-dismissing individuals appear to completely disengage from attachment-related distress when suppressing thoughts of being abandoned by their partner, as evidenced by a lack of galvanic skin response. Most contemporary definitions of compassion include awareness of suffering and a desire to alleviate suffering as key elements of compassion [41, 42]. It follows that for individuals high in attachment avoidance, chronic use of thought suppression which interferes with awareness of distress [31], may inhibit the development of self-compassion. Self-compassion has been shown to buffer against the development of depression [43]. Whilst thought suppression may reduce distress in the short term, should overuse of this strategy interfere with the development of self-compassion, this pattern could lead to increased vulnerability to depression.

### Attachment avoidance, self-compassion and depression

Self-compassion is an adaptive form of self-relating that allows individuals to actively and kindly face difficult life experiences (including negative thoughts and emotions and feelings of vulnerability), without suppressing them or becoming overwhelmed by them [44]. Neff [44] contends that self-compassion is comprised of three interconnected components: self-kindness, common humanity and mindfulness. Self-kindness involves approaching difficult life experiences, such as personal failures, with kindness. Common humanity refers to the recognition that difficulties and struggles are shared human experiences. Mindfulness involves an awareness of thoughts, feelings and sensations which arises through paying attention to the present moment, on purpose, non-judgmentally [45].

Self-compassion is associated with a range of psychological benefits, including improved resilience and improved psychological functioning [46]. In a meta-analysis, self-compassion was reported to be inversely associated with depression, anxiety and stress, with large effect sizes, while being positively associated with improved mental health outcomes [43].

Self-compassion has been theorised to develop in the context of early attachment relationships [47–49]. Neff and McGehee [48] suggest that the way individuals relate to themselves likely reflects their relationships with early caregivers. From this perspective, individuals who experienced unpredictable responses from early caregivers (i.e., at times supportive and at times critical/rejecting) are more likely to be self-critical and self-rejecting, thereby exhibiting lower levels of self-compassion. However, experiences with caregivers who were consistently rejecting and critical can contribute to complex patterns of self-relating, involving both high levels of self-criticism and defensive self-enhancement (e.g., the tendency to inflate self-worth to counter feelings of worthlessness) [50]. These distinct patterns of self-relating have been theorised to influence the development of self-compassion in contradictory ways [49, 51]. For example, the relationship between attachment avoidance and self-compassion may differ depending on whether the individual engages in self-criticism, which would lead to lower levels of self-compassion, or defensive denial and inflating of self-worth, leading to higher levels of self-compassion [52].

Several studies have reported negative associations between attachment avoidance and self-compassion, and between attachment anxiety and self-compassion [49, 53–55]. Moreover, self-compassion has been found to mediate the relationship between attachment avoidance and a range of mental health outcomes, including clinically significant distress (as measured by total scores on the Hospital Anxiety and Depression Scale [54, 56]), anxiety and depression [53] and general mental health difficulties [55].

These studies [53, 54] conclude that low levels of self-compassion may contribute to higher levels of psychopathology, including depression, among individuals with higher levels of attachment avoidance. Moreover, Joeng and colleagues [53] suggest that self-compassion may be an important therapeutic target for individuals with higher attachment avoidance. Nevertheless, the mechanisms underlying the negative association between attachment avoidance and self-compassion, and, in turn, depression remain unclear.

Previous researchers have emphasised the ways in which maladaptive forms of self-relating may contribute to reduced capacity for self-kindness among individuals with higher attachment avoidance [51]. However, we note that there has been limited exploration of factors which inhibit or facilitate the development of self-compassion in individuals who are high in attachment avoidance. The use of deactivating emotion regulation strategies, and specifically thought suppression, has not previously been examined as a mediator between attachment avoidance and self-compassion. Nevertheless, support for this possibility is provided by studies linking attachment, emotion regulation and mindfulness [57]. Furthermore, theory and research has consistently linked self-compassion, emotion regulation and mental health outcomes (see [58] for review) emphasising the interrelationship between emotion regulation strategies and self-compassion in the prediction of distress.

### Attachment avoidance, thought suppression, self-compassion and depression

Self-compassion and emotion regulation are closely linked constructs. Self-compassion requires emotion regulation abilities, including the ability to be aware of, and to tolerate, negative emotions [59]. Several correlational studies have found an inverse relationship between maladaptive emotion regulation strategies (including thought suppression and avoidance coping, associated with deactivation of the attachment system), and self-compassion [44, 46, 60].

Greater use of thought suppression has been considered to be indicative of broader difficulties with emotion regulation, for example, difficulties with accepting emotional experiences and difficulties engaging in adaptive strategies [61, 62]. Moreover, use of deactivating strategies has been shown to contribute to reductions in emotional awareness and emotional clarity [63, 64], both of which are fundamental aspects of self-compassion.

A study by Caldwell and Shaver [57] found that thought suppression and attentional control mediated the relationship between attachment avoidance and mindfulness. Specifically, higher levels of attachment avoidance predicted higher levels of thought suppression which, in turn, predicted lower levels of dispositional mindfulness. Whilst this study employed a cross-sectional design, the authors interpreted this finding as indicating that thought suppression requires cognitive effort and directly interferes with the ability to be aware of present moment experience.

Collectively, the research reviewed suggests that chronic use of thought suppression may undermine self-compassion in a variety of ways. First, self-compassion requires both an awareness of, and active engagement with, negative thoughts and emotions. By chronically suppressing distressing thoughts and emotions, individuals are prevented from actively engaging with these experiences, thereby, inhibiting the development of self-compassion. Secondly, thought suppression distorts emotional experiences, contributing to reduced emotional clarity around emotional states and needs, including the need for comfort, self-soothing and self-compassion. Finally, self-compassion requires volitional attentional effort over and above that required for present moment awareness (e.g., mindfulness). In this way, self-compassion could be seen as actively incompatible with thought suppression—in which cognitive resources are deployed with the aim of keeping thought content outside of awareness.

### Aim and hypotheses

Based on the research reviewed, there is some support for a positive association between attachment avoidance and depression [15] with some null findings opening the possibility that mediating variables may be involved (see [11] for review). Deactivating emotion regulation strategies and self-compassion have both been identified as possible mediators of this relationship [17, 53]. However, previous research has yielded inconsistent findings regarding the mediating effects of deactivating strategies [17]. In contrast, self-compassion has been consistently identified as mediator between attachment avoidance and a range of mental health outcomes, including depression [53–55]. We note, however, that previous research has not clarified the causal mechanisms contributing to the relationship between attachment avoidance and self-compassion. Based on reviewed theory and research [46, 57], higher levels of thought suppression may be one mechanism underlying the negative association between avoidant attachment and self-compassion. Specifically, higher levels of attachment avoidance would be expected to lead to greater use of the deactivating strategy of thought suppression, which may in turn inhibit the development of self-compassion by blocking access to, and engagement with, distressing thoughts. Therefore, higher levels of thought suppression would be expected to be associated with lower levels of self-compassion, with thought suppression and self-compassion being positively and negatively associated with depressive symptoms, respectively. The current study explored this possibility but also extended simple mediation models, by examining the possibility that thought suppression and self-compassion might work sequentially to explain the positive association between attachment avoidance and depression. Therefore, the aim of the current study was to examine this serial mediation model by proposing a theoretically based hypothetical causal chain, in which higher levels of attachment avoidance lead to higher levels of thought suppression, higher levels of thought suppression lead to lower levels of self-compassion, and lower levels of self-compassion, in turn, lead to higher depressive symptoms.

Based on the literature reviewed above, the following hypothesis was formulated and tested:

Thought suppression (mediator 1) and self-compassion (mediator 2), in serial, will mediate the positive relationship between attachment avoidance (IV) and depression (DV).

## Method

### Power analysis

The present study’s required sample size was determined by comparing the results of an *a priori* statistical power analysis (Faul et al., 2009) and two commonly used conventions [65, 66]. The largest number of each of the three analyses was used as the basis for the present study’s sample size. An *a priori* power analysis was undertaken using G*Power [67]. The F test family for linear multiple regression was selected, as being the closest analysis to mediation within G*Power. Power was calculated based on the medium effect size (*f*^2^ = 0.15) recommended for multiple regression analyses [68], three predictor variables, a target power of .80 and an alpha of .05. This process recommended a minimum sample size of 77 participants. Two commonly used conventions for calculating the number of participants for mediation suggested a sample size of 74 participants (*N* = 50 + 8*K*) for testing multiple correlations and a sample size of 107 participants (*N* = 104 + *K*) for testing partial correlations, where *K* = number of predictors and *N* = number of participants [65]. Based on the results of the power analysis, and the two conventions, the recommended minimum number of participants for the present study was 107.

### Participants

Data collection was discontinued when the required minimum sample was reached. A total of 164 participants commenced the questionnaire and 149 participants completed all questions. Only data from completed questionnaires was used in the analysis. The same participant appeared to have completed the survey twice, based on two case numbers with the same age, gender, education level, relationships status and IP address. Thus, one of these cases was deleted. The completion rate was 90.85%. An implied consent procedure was used, wherein consent was implied by participants clicking on the “proceed” button to commence the survey having read the Information for Participants.

The final sample of 148 participants consisted of 107 females (72.3%) and 41 males (27.7%). The mean age, based on 147 participants (one participant’s age was missing), was 34.62 years (*SD* = 13.46), with ages ranging from 18 to 78 years. The majority of the sample (i.e., 73%) reported being in a romantic relationship, 25% of the sample reported being single, and 2% reported never having been in a romantic relationship. The sample was made up of members of the general public recruited via social media and UNE first year psychology students recruited from the UNE Psychology Research Participant pool.

The depression sub-scale of the Depression, Anxiety, and Stress Scale-21 (DASS-21) [69] yielded a mean score of 10.50 (*SD* = 10.27), which was within the mild (non-clinical) range according to DASS-21 cut-offs. However, the sample showed a range of severity symptoms (range: 0–42); that is, 58% were in the normal range (i.e., 0–9), 6.8% reported mild symptoms (i.e., 10–13), 19.6% reported moderate symptoms (i.e., 14–20), 4.7% reported severe symptoms (i.e., 21–27), and 10.8% reported extremely severe symptoms (i.e., 28+).

### Materials

Participants completed a brief demographic questionnaire in which they were requested to provide their age, gender, relationships status, and level of education.

### Experiences in Close Relationships-Revised (ECR-R) [70]

Attachment avoidance and attachment anxiety were measured using the Experiences in Close Relationships-Revised questionnaire [70]. The ECR-R is a 36-item self-report scale, consisting of two 18-item sub-scales: avoidant attachment sub-scale and anxious attachment sub-scale. Participants indicate their agreement with each of the statements on a seven-point Likert response scale ranging from 1 (strongly disagree) to 7 (strongly agree). Example items from the avoidant sub-scale are “I prefer not to be too close to romantic partners” and “I find it difficult to depend on romantic partners.” A person’s attachment avoidance score is calculated by averaging the total score of all attachment avoidance scale items. Scores on both sub-scales range from 1 to 7, with higher scores indicating higher levels of attachment avoidance or attachment anxiety, respectively. The ECR-R has previously been shown to have excellent internal consistency [70]. In the present study, Cronbach’s alpha for the avoidant sub-scale was .95, and for the anxious sub-scale was .94, thus, demonstrating excellent internal consistency.

### Depression, Anxiety and Stress Scale-21. (DASS-21) [69]

Depression was measured using the depression subscale of the DASS-21 [69]. The depression sub-scale measures symptoms of depression including low mood, anhedonia, hopelessness, self-depreciation, reduced interest/involvement in activities, and inertia [69]. Participants are asked to respond to each of the seven items, by indicating how much each of the statements applied to them over the last week. The response scale ranges from 0 to 3, where 0 is “Did not apply to me at all”, 1 is “Applied to me to some degree, or some of the time”, 2 is “Applied to me to a considerable degree or a good part of the time” and 3 is “Applied to me very much or most of the time.” Example items from the depression sub-scale include “I couldn’t seem to experience any positive emotion at all” and “I felt I had nothing to look forward to”. Total depression scores are calculated by summing each of the sub-scale items and multiplying by two. Total depression scores range from 0–42, with higher scores indicating greater depressive symptomology. The DASS-21 is based on a dimensional approach to depression, with cut-off scores indicating the following severity levels: normal (0–9), mild (10–13), moderate (14–20), severe (21–27) and extremely severe (28+). The DASS-21 has previously been shown to have satisfactory reliability and the depression subscale has exhibited excellent reliability [69]. Cronbach’s alpha for the current study was .93, which indicated excellent internal consistency.

### White Bear Suppression Inventory (WBSI) [27]

Thought suppression was examined using the White Bear Suppression Inventory [27]. The WBSI is a 15-item self-report measure, which assesses the habitual use of thought suppression. Schmidt et al. [71] investigated the psychometric properties of the WBSI, identifying two sub-scales: the intrusion sub-scale and the suppression sub-scale. The current study utilised the nine-item suppression sub-scale as identified by Schmidt et al. [71]. This sub-scale consists of a five-point Likert response format ranging from 1 (strongly disagree) to 5 (strongly agree). Example items are “I have thoughts that I try to avoid” and “I always try to put problems out of my mind.” Total scores are calculated by summing scores on each of the nine items. Total scores range from 9 to 45 with higher scores indicating higher thought suppression. The WBSI suppression sub-scale has previously demonstrated good internal consistency, with a Cronbach’s alpha of .86 [57]. Cronbach’s alpha for the present study was .88, thus, also indicating good internal consistency.

### Self-Compassion Scale (SCS) [44]

Self-compassion was measured using the Self-compassion scale [44]. The SCS is a 26-item measure comprised of the following six sub-scales: self-kindness (e.g., “I try to be loving towards myself when I’m feeling emotional pain”), self-judgement (e.g., “When times are really difficult, I tend to be tough on myself”), common humanity (e.g., “I try to see my failings as part of the human condition”), isolation (e.g., “When I fail at something difficult, I tend to feel alone in my failure”) mindfulness (e.g., “When something upsets me, I try to keep my emotions in balance”), and over-identification (e.g., When I’m feeling down I tend to obsess and fixate on everything that’s wrong”). Participants respond to each item of the SCS by indicating how frequently their behaviour is consistent with the stated behaviour. The SCS’s five-point Likert scale ranges from 1 (almost never) to 5 (almost always). A total self-compassion score is achieved by reverse scoring the negative subscale items (e.g., self-judgement, isolation, and over-identification) and then calculating the grand mean of all six sub-scale means [44]. Higher self-compassion scores indicate higher levels of self-compassion. Researchers can analyse their data by using individual sub-scale scores or by calculating a total self-compassion score [44]. Previous research has demonstrated excellent internal consistency and reliability of the total SCS [44]. Cronbach’s alpha for the present study was .94, which indicated excellent internal consistency.

### Procedure

The present study was undertaken following ethics approval from the University of New England’s (UNE) human research ethics committee (approval number: HE18:101). Participants were recruited from the general population via the online social media site Facebook and via the UNE research participation page. UNE students enrolled in introductory psychology units were able to claim course credit in exchange for participation in the study. The study’s specified inclusion criteria included being over 18 years of age and being fluent in written English.

Participants responded to the study invitation by clicking on a link, which directed participants to the online study questionnaire. Participants were first provided with a brief overview of the study, and then provided implied consent by choosing to proceed. Next, participants answered a series of demographic questions followed by a randomised sequence of the ECR-R, DASS-21, WBSI and SCI. The present study’s composite questionnaire included a final debrief, which provided an outline of the study’s aims and hypotheses. Participation took approximately 20 minutes.

### Statistical analyses

**Fig 1:**
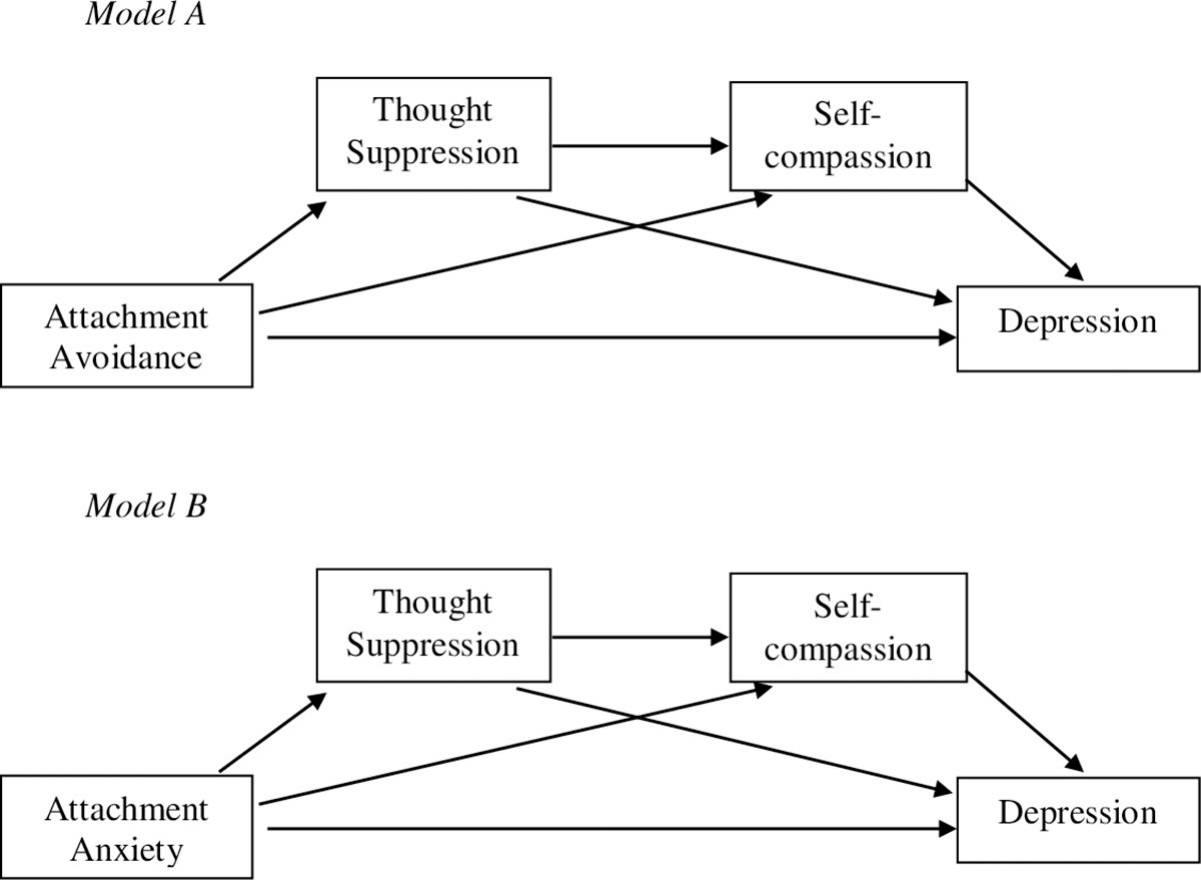
Mediation models. Model A and Model B were assessed using Hayes' PROCESS model six (2018), evaluating thought suppression and self-compassion as mediators of the relationship between attachment avoidance and depression and the relationship between attachment anxiety and depression respectively.

All study data was first analysed using SPSS Version 25 [72]. Kolmogorov-Smirnov tests were conducted to test the assumption of normality [73], which revealed a violation pertaining to the depression variable. In addition, a visual inspection of scatterplots revealed what appeared to be a violation of the assumption of linearity for the following couplings: attachment avoidance and thought suppression, attachment avoidance and depression, and attachment avoidance and self-compassion. Consequently, Spearman’s Rho was used instead of Pearson’s product-moment to assess the relationships between variables [73]. Subsequently, serial mediation analyses were performed using model six of Hayes’ PROCESS (Version 3.5) macro [74], in order to run the planned analysis of mediation model A and the post-hoc analysis of mediation model B, outlined in Fig 1. The models were examined using 10,000 bootstrapped samples, with a random number seed (in this case, 42) to allow for repeated bootstrapping across all analyses, as recommended by Hayes [75]. We note that bootstrapping is a non-parametric statistical procedure and, thus, is distribution-free [75]. In the hypothesised model (Model A), attachment avoidance was entered as the independent variable (IV), depression was entered as the dependent variable (DV), thought suppression was entered as the first mediator (M_1_) and self-compassion was entered as the second mediator (M_2_). A number of researchers have highlighted the importance of statistically controlling for the association between attachment anxiety and avoidance when measuring attachment with self-report measures (e.g., [76]). Consequently, the hypothesised mediation model was analysed twice; firstly, in the manner described above and, subsequently, with the alternative attachment dimension entered as a covariate in the analysis. A second, post-hoc model (Model B), was then tested based on the results of the planned analysis. Within Model B, attachment anxiety was entered as the IV, with depression, thought suppression and self-compassion entered in the same manner as outlined above. Model B was also run twice, with and without the inclusion of attachment avoidance as a covariate.

Process (Model 6) calculates a test of specific indirect effects through both mediators (in serial) and specific indirect effects through each mediator alone [75]. According to the hypothesised serial mediation models there are four possible pathways linking each of the attachment dimensions to depression. The first indirect pathway is through thought suppression (M_1_). The second indirect pathway is through self-compassion (M_2_). The third indirect pathway is through suppression (M_1_), followed by self-compassion (M_2_), in serial. The final pathway is the direct pathway from each attachment dimension to depression. Models were examined using 10,000 bootstrapped samples by (Hayes, 2018). Bootstrapping is a non-parametric statistical procedure that is not dependent on normal distribution assumptions [75, 77]. The primary assumption for bootstrapping is that the sample is representative of the normal population [75].

## Results

### Correlations

Table 1 provides a summary of means, standard deviations and correlations for each of the study variables. As seen in Table 1, each of the variables evaluated were significantly correlated.

**Table 1:** Spearman’s correlations, means and standard deviations between key study variables (n = 148).

	1	2	3	4	5
1. Attachment Avoidance		.52**	.21*	-.23**	.27**
2. Attachment Anxiety			.26**	-.45**	.40**
3. Thought suppression				-.57**	.44**
4. Self-compassion					-.52**
5. Depression					
*M*	2.80	3.02	46.09	2.94	10.50
*SD*	1.18	1.29	14.31	0.72	10.27

*Note*: All correlations are two-tailed

*p < .05.

**p < .01; *M* = Mean; *SD* = Standard deviation.

### Mediation analysis

#### Model A

Given that previous research (e.g., [76, 78]) has found a significant association between attachment avoidance and attachment anxiety, it may be such that significant associations observed between either attachment dimension and other study variables are an artefact of the association between the attachment dimensions. To rule out this possibility, the mediation model displayed in Fig 1 (Model A) was run twice, with attachment anxiety entered as a covariate in the second iteration of the analysis. In the first iteration of the analysis, the total effect was significant (*b* = 2.67, 95% CI = 1.32 to 4.02, *β* = .31, *p* < .001). The direct effect of attachment avoidance on depression was significant (*b* = 1.60, 95% CI = .39 to 2.80, *β* = .18, *p* = .010). The total indirect effect was also significant (*b* = 1.07, 95% CI = .33 to 1.87, *β* = .12), with a significant serial mediation effect being observed from attachment avoidance via thought suppression and self-compassion to depression (*b* = .39, 95% CI = .05 to .75, *β* = .04). The pathway from attachment avoidance via thought suppression, i.e., attachment avoidance→thought suppression→depression, was not significant (*b* = .21, 95% CI = -.07 to .62, *β* = .02). The indirect pathway via self-compassion, i.e., attachment avoidance→self-compassion→depression was not significant (*b* = .47, 95% CI = -.03 to 1.14, *β* = .05).

In the second iteration of the analysis, when controlling for attachment anxiety, the total model effect was not significant (*b* = 1.23, 95% CI = -.22 to 2.68, *β* = .14, *p* = .097). Table 2 provides a summary of the significance of the individual pathways assessed in Model A, as well as the total, direct and indirect effects of the mediation, whilst controlling for attachment anxiety. The results indicate that the direct (*b* = 1.08, 95% CI = -.24 to 2.39, *β* = .12, *p* = .108) and indirect effects (*b* = .15, 95% CI = -.60 to .85, *β* = .02) were non-significant. The hypothesised relationships represented in Model A were not supported after controlling for attachment anxiety.

**Table 2:** Serial mediation effects of thought suppression and self-compassion on the relationship between attachment avoidance and depression whilst controlling for attachment anxiety (Model A).

Model pathways	*Coefficient b (*β)	*SE*	*t*	*p*	*LL 95%CI*	*UL95%CI*
Att. Avoid. → Thought. Supp	1.03	1.09	.94	.348	-1.13	3.19
Att. Avoid. →Self-Compass	.02	.04	.36	.717	- .07	.10
Thought. Supp → Depression	.10	.06	1.64	.104	- .02	.22
Self-Compass. → Depression	-4.97	1.30	-3.83	< .001**	**-7.53**	**-2.40**
Thought. Supp → Self-Compass.	-.03	.00	-7.70	< .001**	**- .03**	**- .02**
Total Model Effect	1.23 (.14)	.73	1.67	.097	-.22	2.68
Direct Effect	1.08 (.12)	.67	1.62	.108	-.24	2.39
Total Indirect Effect	.15 (.02)	.37			-.60	.85
Att. Avoid. → Thought. Supp → Depression	.10(.01)	.15			-.18	.45
Att. Avoid. →Self-Compass. → Depression	-.08 (-.01)	.21			-.50	.37
Att. Avoid. → Thought. Supp → Self-Compass. → Depression	.13 (.01)	.16			-.20	.46

*Note*. * pathway significant at *p* < .05

** pathway significant at *p* < .001; significant pathways are noted in bold (95% confidence interval does not cross zero). All pathways are unstandardised. Indirect effects were computed using 10,000 bootstrap samples. Unstandardised indirect effects are shown outside parentheses. Standardised indirect effects are shown inside parentheses.

### Post-hoc mediation analysis

#### Model B

Apropos of the findings across the two iterations of Model A reported above, which indicated that shared variance with attachment anxiety was driving the effect initially observed, a second, post-hoc mediation model (depicted in Model B) was also tested. Model B was tested twice, with attachment avoidance entered as a covariate in the second iteration of the analysis. In the first iteration of the analysis, the total model effect was significant (*b* = 3.34, 95% CI = 2.16 to 4.52, *β* = .42, *p* < .001). The direct effect of attachment anxiety on depression was significant (*b* = 1.71, 95% CI = .50 to 2.93, *β* = .22, *p* = .006). The total indirect effect was also significant (*b* = 1.63, 95% CI = .91 to 2.48, *β* = .20). The mediation pathway from attachment anxiety via self-compassion (i.e., the indirect mediating effect of self-compassion) was significant (*b* = .95, 95% CI = .38 to 1.70, *β* = .12). The serial mediation effect from attachment anxiety via thought suppression and self-compassion to depression was also significant (*b* = .36, 95% CI = .12 to .66, *β* = .05). The indirect pathway via thought suppression (i.e., attachment anxiety→thought suppression→depression) was not significant (*b* = .31, 95% CI = -.02 to .80, *β* = .04).

After controlling for attachment avoidance, the total model effect remained significant (*b* = 2.81, 95% CI = 1.49 to 4.14 *β* = .35, *p* < .001), while the direct effect of attachment anxiety on depression was no longer significant (*b* = 1.26, 95% CI = -.07 to 2.59, *β* = .16, *p* = .064). The overall indirect effect was significant (*b* = 1.56, 95% CI = .74 to 2.53, *β* = .20). A significant mediating effect was again found in the pathway via self-compassion, i.e., attachment anxiety→self-compassion→depression (*b* = 1.00, 95% CI = .42 to 1.75, *β* = .13). The hypothesised serial mediation indirect pathway (attachment anxiety→thought suppression→self-compassion→depression) was also significant (*b* = .31, 95% CI = .03 to .67, *β* = .04). Again, the indirect effect via thought suppression was not significant (*b* = .25, 95% CI = -.04 to .75, *β* = .03). The results of the mediation analysis evaluating Model B, whilst controlling for attachment avoidance, are summarised in Table 3.

**Table 3:** Serial mediation effects of thought suppression and self-compassion on the relationship between attachment anxiety and depression whilst controlling for attachment avoidance (Model B).

Model pathways	*Coefficient b (*β)	*SE*	*t*	*p*	*LL 95%CI*	*UL95%CI*
Att. Anxiety → Thought. Supp	2.52	1.00	2.52	.013*	**.54**	**4.49**
Att. Anxiety. →Self-Compass	-.20	.04	-5.03	< .001**	**- .28**	**-.12**
Thought. Supp → Depression	.10	.06	1.64	.104	- .02	.22
Self-Compass. → Depression	-4.97	1.30	-3.83	< .001**	**-7.53**	**-2.40**
Thought. Supp → Self-Compass.	-.03	.00	-7.70	< .001**	**- .03**	**- .02**
Total Model Effect	2.81 (.35)	.67	4.19	< .001**	**1.49**	**4.14**
Direct Effect	1.26 (.16)	.67	1.87	.064	-.07	2.59
Total Indirect Effect	1.56 (.20)	.45			**.74**	**2.53**
Att. Anxiety → Thought. Supp → Depression	.25(.03)	.20			-.04	.75
Att. Anxiety →Self-Compass. → Depression	1.00 (.13)	.34			**.42**	**1.75**
Att. Anxiety → Thought. Supp → Self-Compass. → Depression	.31(.04)	.16			**.03**	**.67**

*Note*.

* pathway significant at *p* < .05

** pathway significant at *p* < .001; significant pathways are noted in bold (95% confidence interval does not cross zero). All pathways are unstandardised. Indirect effects were computed using 10,000 bootstrap samples. Unstandardised indirect effects are shown outside parentheses. Standardised indirect effects are shown inside parentheses.

**Fig 2:**
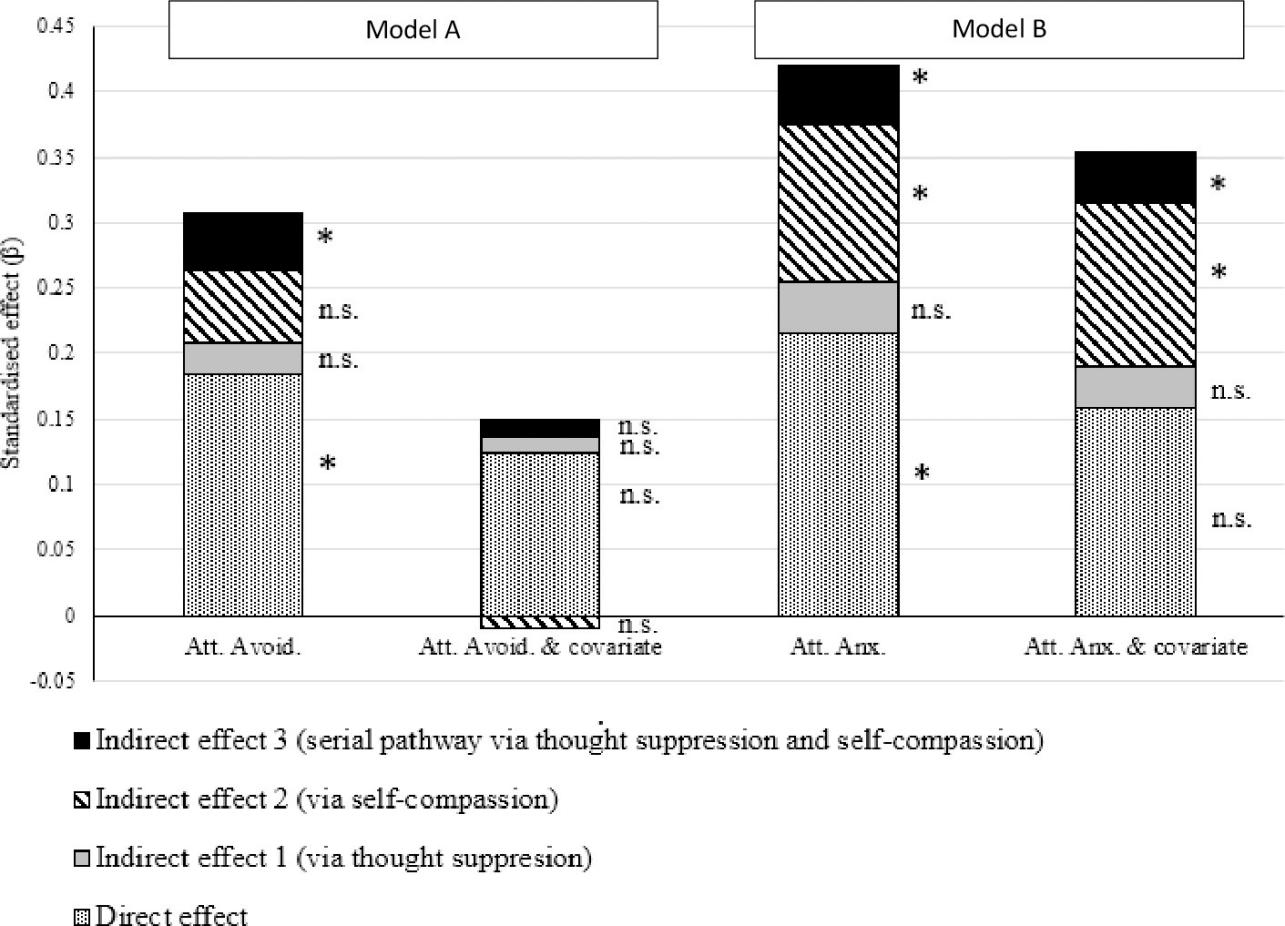
Effect sizes for Model A and Model B. Sizes of direct and indirect effects as indicated by standardized beta weights 03) for Model A (Att. Avoid., Att. Avoid & covariate) and Model B (Att. Anx., Att. Anx. & covariate) without and with the alternative attachment dimension as covariate. *—effect significant as indicated by 95% confidence interval not crossing zero. n.s.- effect non-significant as indicated by 95% confidence interval crossing zero.

The results, therefore, support the serial mediating effects of thought suppression and self-compassion in the relationship between attachment anxiety and depression, after controlling for attachment avoidance, as well as the role of self-compassion as a mediator in this relationship. The results did not support the hypothesised role of thought suppression as an individual mediator in the relationship between attachment anxiety and depression whilst accounting for the other indirect pathways assessed. Similarly, the direct pathway between attachment anxiety and depression was not significant. As recommended by Wen and Fan [79], the size of the standardized direct and indirect effects from each of the reported mediation analyses are summarised in Fig 2.

## Discussion

The present study aimed to examine the potential mediating effects of thought suppression and self-compassion on the relationship between attachment avoidance and depression. The preliminary analysis indicated that attachment avoidance demonstrated a positive association with thought suppression; a negative association with self-compassion; and a positive association with depressive symptomology. The relationship between attachment avoidance and depression was found to be fully mediated by the serial mediation pathway via thought suppression and self-compassion (i.e., the direct effect of attachment avoidance on depression was non-significant when accounting for this indirect pathway). However, after controlling for attachment anxiety, the total, direct and indirect effects evaluated in the mediation model were found to be non-significant. Furthermore, the individual pathways between attachment avoidance and each of the mediators and DV (i.e., thought suppression, self-compassion and depression) were each non-significant. A similar pattern of findings has been reported in previous studies [78, 80], wherein the previously significant relationships between attachment avoidance and other study variables were rendered non-significant once attachment anxiety was included as a covariate. One possible interpretation of this finding is that the observed significant effects in the original model were driven by the variance which is common to both attachment dimensions. Consequently, when this shared variance is removed the hypothesised relationships outlined in mediation Model A were not supported.

Attachment anxiety demonstrated a significant association with thought suppression, self-compassion and depression. In order to verify the above interpretation of the findings of Model A, a second serial mediation model was tested as part of the post-hoc analysis. Model B was identical to Model A, but with attachment anxiety entered as the independent variable. Within Model B, the relationship between attachment anxiety and depression was found to be partially mediated by thought suppression and self-compassion. Specifically, the indirect pathway via self-compassion and the hypothesised serial mediation pathway via thought suppression and self-compassion were found to be significant, as were the total model and direct effects. After controlling for attachment avoidance, these indirect effects, and the total model effect, remained significant but the direct relationship between attachment anxiety and depression was found to be non-significant, therefore, indicating that the total model and indirect effects may not be attributed to shared variance between the attachment dimensions.

In summary, extant literature and theory suggested a sequential pathway between attachment avoidance, suppression, self-compassion and depression, which we tested in Model A. Our findings suggest that, whilst Model A was significant, the relationships are largely driven by shared variance between attachment anxiety and attachment avoidance, as these effects were non-significant when covarying for attachment anxiety. The results of the present study provide provisional support for the hypothesised relationships outlined in Model B, which suggest that thought suppression and self-compassion, in serial, mediate the relationship between attachment anxiety and depression. This is a novel finding which has not previously been investigated.

In interpreting our findings, we firstly note that the results of the present study broadly support the idea that attachment anxiety is associated with the use of maladaptive cognitive emotion regulation strategies, which, in turn, are associated with levels of depressive symptoms. The findings suggest that both attachment anxiety and the use of maladaptive emotion regulation strategies are inversely related to self-compassion. Specifically, as attachment anxiety increases, so too does the use of strategies to avoid threatening inner experiences increases (e.g., suppression of thoughts pertaining to self-worth, self-criticism, abandonment, negative relationship experiences and/or imagined future events). As self-compassion inherently involves engaging with one’s own vulnerability or distress with equanimity, it makes conceptual sense that this mode of self-relating is somewhat incompatible with strategies of avoiding inter-personal vulnerability and suppressing distressing thoughts. The negative association between thought suppression and self-compassion in the present study is consistent with previous findings of an inverse relationship between thought suppression and mindfulness [57] and suggests that high levels of suppression may limit the ability to engage in self-compassionate responses. We acknowledge that the temporal sequence of mediators cannot be adequately tested in a cross-sectional design (see limitations below). However, these findings at least provide evidence of a degree of incompatibility between suppression and self-compassion, as they operate in the relationship between attachment orientation and depression. Finally, these results support the contention that self-compassion buffers against vulnerability to depressive symptoms. The negative association found between self-compassion and depression is consistent with a previous meta-analysis which yielded robust evidence of an inverse relationship between self-compassion and psychopathology (including depression, anxiety and stress) [43].

Secondly, self-compassion emerged as a significant standalone mediator of the relationship between attachment style and depression in both models, consistent with the findings of Joeng et al. [53]. In contrast, the suppression to depression pathway was not significant in either model, unless self-compassion was added as a second mediator of this relationship. We note that thought suppression has been argued to contribute to depression under conditions of high stress only [29]. However, these findings suggest that, in the context of insecure attachment, use of thought suppression predicts vulnerability to depression only to the extent that it also interferes with self-compassionate responses. Given the mutual exclusivity noted above, this is more likely under conditions of high suppression, which are more likely to occur under conditions of high stress. This pattern of findings fits with our earlier interpretation of Malik et al.’s [17] mixed findings regarding suppression as a mediator of the insecure attachment to depression relationship, and support this study’s aims in considering both maladaptive emotion regulation strategies (suppression) and adaptive coping (self-compassion) in predicting overall vulnerability to depressive symptomology.

Finally, as previously stated, the findings of the present study did not support the hypothesised relationships outlined in mediation Model A, after controlling for attachment anxiety. Specifically, once shared variance between attachment avoidance and attachment anxiety was taken into account, the mediation analysis indicated that attachment avoidance did not demonstrate a significant relationship with thought suppression, self-compassion or depressive symptoms. This finding may assist with making sense of inconsistent findings regarding the relationship between attachment avoidance, emotion regulation strategies and depression reported by Malik et al. [17]. One possible interpretation is that inconsistent findings across studies may be reflective of underlying differences in *attachment anxiety* within the sample, which was not accounted for in the analyses (i.e., where significant relationships emerged between attachment avoidance, dysfunctional emotion regulation strategies and depression, these findings may have been driven by elevated levels of attachment anxiety in the sample whereas non-significant results may have reflected lower levels of attachment anxiety in the sample).

Fundamentally, our results are consistent with the view that attachment anxiety and avoidance are not distinct, orthogonal constructs, but rather, are overlapping to a degree, as suggested by Cameron et al. [76]. In practice, most people with an insecure attachment style will present with some degree of elevation across both dimensions. In any given relational context, one dimension will emerge as being relatively more elevated than the other. It may, therefore, be important to resist conceptually splitting the dimensions, and to consider individuals as predominantly manifesting one or other dimension, in view of the finding that there is considerable shared variance across the dimensions. This interpretation is consistent with the findings of Cameron et al. [76], and support the authors’ recommendation to account for shared variance in any analysis in order to determine which adult attachment dimension emerges as the significant predictor of any given outcome or mediating process.

Similarly, the finding that attachment anxiety emerged as the more robust predictor of variables considered in this study was somewhat unexpected based on the literature reviewed. Thought suppression is typically considered a deactivating strategy, and has most commonly been associated with attachment avoidance [30–32]. From a theoretical perspective, attachment avoidance is more likely to be associated with the suppression of thoughts related to vulnerability and the need for connection, since these would typically activate the attachment system, cuing people to reach out to others for support [2]. It may be that attachment anxiety is associated with suppression of thoughts relating to the need for self-assertion and independence, since these thoughts would conflict with the felt need for proximity and connection. This interpretation is supported by the findings of a recent study examining emotion-specific suppression and attachment dimensions [81]. Attachment anxiety was associated with greater suppression of anger and dysregulation of sadness and worry, whereas attachment avoidance was associated with greater suppression of sadness and worry, and greater dysregulation of anger. This suggests that similar dysfunctional emotion regulation strategies may be recruited for different purposes, depending on which attachment dimension is more influential for an individual in a given relational context.

### Limitations and suggestions for future research

While it is permissible to perform mediational analyses using cross-sectional data [75], and indeed, an appropriate first step in exploring our hypothesised serial mediation model is to employ a cross-sectional design (e.g., [82]), we are precluded from making any causal claims regarding our mediation model. Consequently, it would be prudent for future research to test our serial mediation model using experimental and/or longitudinal designs. Furthermore, we emphasise that the temporal sequence of the present study’s mediators cannot be verified with cross-sectional data. We note that it has also been demonstrated that higher levels of self-compassion predict lower levels of suppression [58], suggesting a reversed temporal sequence to the models tested in this study. This also raises the possibility that the relationship between candidate mediators is bi-directional. Whilst this possibility cannot be tested within a cross-sectional design, the significant results for Model B obtained here indicate that it would be appropriate to employ a longitudinal and/or experimental design to test the hypothetical causal chain within a future study. Additionally, future research may test the potential bi-directionality of self-compassion and thought suppression using a cross-lagged effects model (see, e.g., [83]). Specifically, one could assess whether self-compassion quantified at t1 has an effect on thought suppression quantified at t2 and whether thought suppression quantified at t1 has an effect on self-compassion quantified at t2. Results supporting the two aforementioned effects would indicate a bi-directional effect.

This study was based on a sample of psychology undergraduates and members of the general population. In order to maintain strict participant confidentiality, no personally identifying information was collected. Participant status as a student/nonstudent was not recorded and so cannot be reported. As such, it is not possible to determine the degree to which the findings may be generalised to the wider population. Linked to this, the sample had an uneven gender distribution, with more females than males taking part in the study. Future studies with more evenly distributed gender samples could be undertaken to determine whether results generalise across both genders. Given that gender has been shown to influence levels of self-compassion [84], suppression [85] and depression levels [86], it is important that gender effects also be examined in future studies.

The distribution of depressive symptoms in the sample was positively skewed, which is consistent with base rate depressive symptoms in the general population [9]. However, this skewness limits the extent to which the study’s findings may apply within clinical populations. As such, one important avenue for further research would be to replicate the present study within a clinical population seeking treatment for depression. Such a study would have important implications for treatment, since there are established evidence-based interventions which target the use of adaptive emotion regulation strategies and exercises to develop self-compassion [87, 88].

A further limitation is the study’s sole reliance on self-report measures. Self-report measures are subject to bias and may not provide an accurate assessment of study variables [28]. These biases may be especially apparent for individuals with high levels of attachment avoidance, whose use of deactivating strategies and avoidant defences may mean that they lack clarity about emotional states and/or defensively underreport negative emotional experiences [34, 44]. Study results could be strengthened by utilising a range of different measurement modalities, including physiological, behavioural, and observation measures and clinical interviews.

## Conclusion

The present study was the first to examine thought suppression and self-compassion as serial mediators of the relationship between attachment orientation and depression. The results of the present study indicate that thought suppression followed by self-compassion (in serial) may be important mechanisms that contribute to the positive relationship between attachment anxiety and depression. Our findings support the contention that thought suppression and self-compassion operate in opposing directions in the relationship between attachment anxiety and depressive symptoms. Furthermore, our data suggest self-compassion is a significant standalone mediator of this relationship, whereas suppression is not. One plausible interpretation of the present findings is that thought suppression influences vulnerability to depression through its negative impact on the development of self-compassion. This hypothetical causal chain could be tested in a follow-up longitudinal study.

Secondly, when considering attachment dimensions independently, both serial mediation pathways were significant. However, when accounting for shared variance between attachment dimensions, attachment anxiety emerged as the more robust predictor of vulnerability to depression through the thought suppression to self-compassion sequence. This somewhat unexpected finding indicates the need for future studies to include both attachment dimensions within any research design, consistent with the recommendations of Cameron et al. [76]. Our findings also have implications for the conceptualisation of attachment domains as overlapping rather than distinct, which is consistent with the correlation typically observed between the dimensions [89]. Correspondingly, our findings indicate that attachment anxiety significantly predicts use of thought suppression, although this is commonly conceptualised as a deactivating strategy linked with attachment avoidance [90]. Our data cannot speak to the issue of whether thought suppression is recruited for different purposes, depending on which attachment domain is relatively more influential. This possibility is worthy of further exploration, as the present findings fit with emerging evidence of differential profiles of emotion-specific suppression across the attachment domains [81].
